# Broadening the spectrum of controls for skin biopsy in painful neuropathies: spondylotic cervical myelopathy patients with painful feet

**DOI:** 10.1002/brb3.444

**Published:** 2016-02-16

**Authors:** Ivana Kovalova, Eva Vlckova, Josef Bednarik

**Affiliations:** ^1^ Department of NeurologyUniversity Hospital BrnoJihlavska 2062500BrnoCzech Republic; ^2^ Central European Institute of TechnologyMasaryk UniversityBrnoCzech Republic

**Keywords:** Cervical spondylotic myelopathy, epidermal nerve fibers, neuropathic pain, peripheral neuropathy, skin biopsy

## Abstract

**Purpose:**

Intraepidermal nerve fiber density (IENFD) is useful in the evaluation of small‐fiber neuropathy (SFN). Recent guidelines recommend extending the spectrum of controls for IENFD assessment by evaluation of patients whose clinical picture mimics that of SFN. The aim of this study was to broaden the spectrum of IENFD controls by the assessment of patients with cervical spondylotic myelopathy (CSM) and painful feet.

**Methods:**

Evaluation of IENFD from skin biopsy samples and quantitative sensory testing (QST) were performed in a cohort of 14 CSM patients (eight men, median age: 58; range: 46–63 years), with painful feet, exhibiting no clinical or electrophysiological signs of large‐fiber polyneuropathy, and no risk factors for peripheral neuropathies.

**Results:**

Quantitative sensory testing abnormalities were found in all but two of the CSM patients (86%), while the IENFD values were within reference range. The mean IENFD value (6.87 ± 2.78 fibers/mm) did not differ from that of an age‐ and sex‐matched cohort of healthy volunteers (7.97 ± 2.21 fibers/mm, *P* > 0.05).

**Conclusions:**

The study confirmed normal skin biopsy findings in patients with CSM as one of the clinical conditions mimicking SFN and provided further support for the use of IENFD assessment in case of suspicion of SFN.

## Background

In recent years, skin biopsy with assessment of intraepidermal nerve fiber density (IENFD) has increasingly been used in the evaluation of small‐fiber neuropathy (SFN) (England et al. 2009; Lauria et al. 2010b). An American Academy of Neurology, American Association of Neuromuscular and Electrodiagnostic Medicine (AANEM), and American Academy of Physical Medicine and Rehabilitation evidence‐based review issued a Level C recommendation that supports skin biopsy in the diagnostic evaluation of SFN (England et al. 2009), while the European Federation of Neurological Societies/Peripheral Nerve Society Guideline (Lauria et al. 2010b) stated that decreased IENFD reliably indicates the presence of SFN (level A recommendation). Both recommendations, however, emphasize the need to broaden the spectrum of controls for IENFD assessment by the evaluation of skin biopsy in patients whose clinical picture mimics that of SFN (Lacomis 2002).

A clinical picture similar to that exhibited by SFN, with positive sensory symptoms in the lower extremities together with clinical or quantitative sensory testing (QST) abnormities in this distribution, may also be found in certain other clinical conditions, particularly those affecting central somatosensory pathways; these may be confused with SFN. Recently, this emerged in a study of patients with multiple sclerosis and restless legs syndrome (Herrmann et al. 2010). Another disease that may present with sensory symptoms and signs in the lower legs is cervical spondylotic myelopathy (CSM) (Yoshiyama et al. 1995). Sensory symptoms in the lower extremities were included within the original Japanese Orthopaedic Association (JOA) scale (Yonenobu et al. 2001) used in CSM evaluation, but not in the modified (mJOA) score (Benzel et al. 1991).

The aim of this study was to extend the range of controls for skin biopsy and IENFD evaluation in patients with CSM and positive sensory symptoms in the lower legs, a condition resembling SFN.

## Methods

A large cohort of 244 CSM patients, followed up by the Department of Neurology of the University Hospital, Brno, was screened for the presence of positive sensory symptoms in the lower legs mimicking SFN. Such symptoms were described by a total of 42 patients from this cohort. These were clinically examined, screened for known risk factors for peripheral neuropathies, and given standard nerve conduction studies. Nineteen of these patients fulfilled the following inclusion criteria: (1) pain intensity of at least 3 as assessed by a numerical rating scale ranging from 0 to 10, in which 0 represented “no pain” and 10 “the worst pain I can imagine”; (2) no risk factors for peripheral neuropathies in the medical history (in particular, diabetes mellitus, alcohol abuse, uremia, thyroid disorders, malignancy, or exposure to toxins or medication associated with neuropathy); (3) normal or increased deep tendon reflexes in the lower legs; (4) normal nerve conduction studies in the lower extremities, to exclude large nerve‐fiber involvement in this distribution; and (5) absence of other diseases or conditions leading to foot pain (i.e., plantar fasciitis, tarsal tunnel syndrome, osteoarthritis, peripheral vascular disease). Finally, only 14 patients fulfilled inclusion criteria and were willing to participate (eight men, six women, median age: 58; range: 46–63 years – see Table 1). The study was approved by the local Ethics Committee and written informed consent was obtained from all the participants.

**Table 1:** Demographic data, mJOA score, evoked potentials, skin biopsy findings, and quantitative sensory testing in CSM patients

Patient no.	Sex	Age	mJOA	MEP	SEP	IENFD	QST abnormality	
							Gain	Loss
1	M	46	17	N	N	5.9^a^^,^ b	MPT, MPS	MDT, VDT
2	M	55	16	N	N	4.3^a^	PHS	CDT, TSL, MDT
3	F	60	14	C	N	7.4^a^^,^ b	0	0
4	F	59	17	N	C	14.7^a^^,^ b	0	0
5	M	61	17	N	N	5.0^a^^,^ b	PHS, MPT, MPS	CDT, TSL
6	F	55	13	C	N	4.3^a^	PHS	MPT
7	M	62	16	N	N	7.5^a^^,^ b	PHS, MPT, HPT	0
8	M	51	17	N	N	6.3^a^^,^ b	PHS, WUR	CDT, TSL, MDT
9	F	61	15	N	N	4.7^a^^,^ b	0	WDT, MPT
10	M	53	17	N	C	6.4^a^^,^ b	0	WDT, TSL
11	F	63	16	N	N	5.0^a^^,^ b	PPT	CDT
12	F	57	16	N	C	7.2^a^^,^ b	0	CDT, WDT, TSL, MDT
13	M	62	14	N	C	6.4^a^^,^ b	0	PPT, MDT
14	M	50	17	N	N	11.2^a^^,^ b	PHS	CDT, WDT, TSL

CSM, cervical spondylotic myelopathy; mJOA, modified Japanese Orthopaedic Association (JOA) scale (Benzel et al. 1991); MEP, motor‐evoked potentials; SEP, somatosensory‐evoked potentials; N, normal finding of MEP or SEP; C, central conduction abnormality in MEP/SEP attributed to possible cervical spinal cord lesion; IENFD, intraepidermal nerve fiber density; QST, quantitative sensory testing; CDT, cold detection threshold; WDT, warm detection threshold; TSL, thermal sensory limen; HPT, heat pain threshold; PPT, pressure pain threshold; MPT, mechanical pain threshold; MPS, mechanical pain sensitivity; WUR, wind‐up ratio; MDT, mechanical detection threshold; VDT, vibration detection threshold; PHS, paradoxical heat sensation.

a IENFD value within normal range, after Lauria et al. (2010a,b).

b IENFD value within normal range, after Bursova et al. (2012).

All the individuals included fulfilled the diagnostic criteria for cervical spondylotic myelopathy based on clinical symptoms and signs of cervical cord dysfunction or lesion due to spondylotic cervical cord compression, documented by magnetic resonance imaging (MRI) (Emery 2001). Severity of clinical disability was graded by means of mJOA score (Benzel et al. 1991).

Standard short‐latency SEPs from the median and the tibial nerves were elicited with electrical stimulation of mixed nerves at the wrist and the ankle, and recorded using a Nicolet four‐channel Viking II unit.

Motor evoked potencials (MEPs) were elicited using a MAGSTIM 200 magnetic stimulator and circular 90‐mm (type 9784) stimulating coil with a peak magnetic field strength of 2.0 T. On‐line data acquisition was performed using a Dantec Keypoint electromyograph. MEPs were elicited by means of transcranial and root magnetic stimulation, and recorded from abductor digiti minimi and abductor hallucis muscles on both sides.

The details of the testing procedures of both SEPs and MEPs were previously published in detail (Bednarík et al. 1999), including the criteria for the definition of central conduction abnormality attributed to possible cervical spinal cord lesion.

Quantitative sensory testing examination in the lower legs was performed, following the standardized protocol of the German Research Network on Neuropathic Pain (Deutscher Forschungsverbund Neuropathischer Schmerz, DFNS) (Rolke et al. 2006) and using its standard recommendations. The Czech version of the instructions used for this purpose has recently been validated and the applicability of published reference values (Magerl et al. 2010) has been confirmed for the Czech population (Srotova et al. 2015). All the tests were performed on the dorsum of the foot with the exception of PPT, which was tested on the sole. The symptoms were symmetrical in all the 14 cases. One‐sided QST testing was therefore performed on the right side.

Skin‐punch biopsy samples were taken from the distal calf, approximately 10‐cm above the right lateral malleolus. The details of specimen removal and staining techniques have been widely published (Vlckova‐Moravcova et al. 2008; Skorna et al. 2015) and follow standard recommendations (Lauria et al. 2010b). In brief, after fixation in 4% phosphate‐buffered paraformaldehyde (pH 7.4) and cryoprotection in 10% sucrose, frozen sections of 50 *μ*m thickness were cut and immunostained with rabbit polyclonal antibodies to human PGP‐9.5 (1:200; Ultraclone, Wellow, UK, Ultraclone Cat. RA95101, RRID: AB_2313685) as primary antibody, and goat anti‐rabbit IgG labeled with fluorescein probe as secondary antibody (1:100; Chemicon, Temecula, CA). The intraepidermal nerve fibers were counted manually at ×630 magnification using a Leica DMLB microscope (Leica Microsystems GmbH, Wetzlar, Germany). The epidermal length of the skin section was measured with calibrated software (ImageJ, NIH Image, http://rsb.info.nih.gov/ij/, 1.42p, RRID:SCR_003070). The average intraepidermal nerve fiber density (IENFD) per millimeter of epidermal length was calculated according to current guidelines (Lauria et al. 2010b). The entire epidermal length of three nonadjacent sections was evaluated in each patient. All the samples were counted by a single observer unaware of the clinical data (IK) and re‐evaluated by another (EV) to ensure reliability. The results were evaluated using our own laboratory normative values, obtained from a smaller sample of individuals by immunofluorescence (Bursova et al. 2012) as well as published reference values based on results from a large cohort of normal individuals using bright‐field immunohistochemistry (Lauria et al. 2010b).

## Results

All the patients included in the study showed only mild and predominantly sensory clinical impairment (mainly sensory loss, in upper and lower extremities, but without pronounced lack of stability when walking) which corresponds with high values of mJOA score in all the cases (Table 1). Clinical presentation of CSM thus corresponds with the “pseudopolyneuropathic pattern” of distal sensory involvement in lower extremities, which mimics the SFN (in part in combination with plurisegmental sensory loss in one or both upper extremities probably due to lesions of dorsal horns at the cervical level).

Central conduction abnormalities in somatosensory or motor‐evoked potentials were found in a small proportion of the cases due to associated lesions of the corticospinal tract and dorsal columns at the cervical level.

Quantitative sensory testing protocol revealed numerous sensory abnormities in the lower extremities in most of the participants (Table 1). Only two of the 14 patients (14%) had no QST abnormity. In one patient (7%), only gain abnormities were revealed, while four patients (29%) displayed only loss abnormalities. Most frequently, a combination of both positive and negative sensory signs emerged upon QST examination (seven patients, i.e., 50%) (Table 1).

The IENFD values for all 14 patients were within the range of age‐ and sex‐related reference values when using the reference data from the worldwide normative study (Lauria et al. 2010a). When using our own normal data, IENFD values were borderline in two of the patients, ranging between *x*‐2 SD and *x*‐2.5 SD of the reference data set, while all the other values did not exceed normal range (Table 1). On a group basis, however, mean IENFD values in the CSM group were not significantly different (6.87 ± 2.78 fibers/mm of epidermal length) from the cohort of healthy volunteers matched for age and gen‐der with this study (7.97 ± 2.21 fibers/mm, *P* > 0.05) (Vlckova‐Moravcova et al. 2008; Bursova et al. 2012).

## Discussion

This prospective study confirmed normal skin biopsy findings in patients with cervical spondylotic myelopathy as one of the clinical conditions that can mimic small‐fiber neuropathy. Such a finding provides further support for the use of IENFD evaluation from skin biopsy in the diagnosis of SFN. Our study thus confirms recommendations for future research on this topic, tallying with recent reviews and guidelines (England et al. 2009; Lauria et al. 2010b).

In CSM, sensory pathways are frequently involved at the cervical level, and pseudopolyneuropathic patterns of sensory dysfunction in the lower extremities may be found in a significant proportion of CSM patients (Yoshiyama et al. 1995). In our group of patients, positive sensory symptoms, including pain, were less frequent than negative symptoms and signs, but not exceptional. In our large cohort of 244 CSM patients, such positive symptoms were found in 42 individuals (i.e., 17%). As well as clinical evidence of abnormal thermal and/or pain sensitivity, most of the patients included in our study also exhibited multiple QST abnormalities, usually in both loss and gain directions. Our findings are thus in agreement with the assumption that positive as well as negative sensory symptoms and signs in the lower extremities are not rare in CSM patients. Cervical spondylotic myelopathy may thus parallel some of the criteria used for SFN diagnosis and may be confused with this condition, thus constituting a suitable control group for IENFD evaluation. This study therefore successfully broadens the spectrum of controls for skin biopsies in the diagnosis of peripheral neuropathies, in similar fashion to a further, recently published study using different control groups of patients (Herrmann et al. 2010).

Predominantly, sensory involvement in our selected group of CSM patient is documented by quite high values of mJOA score. SEPs and/or MEPs showed the abnormality only in a small proportion of the CSM patients. These results also support only the mild and predominantly sensory (i.e., pseudopolyneuropathic) pattern of involvement mimicking SFN, which is supposed to result from spinothalamic tract dysfunction and thus is not reflected by any abnormality in SEP examination. Central conduction abnormality of somatosensory‐evoked potentials results from the lesion of dorsal spinal column, while dysfunction of the dorsal horn in the lower cervical spine leads to segmental abnormality of N13 wave (only).

The main limitation of this study is sample size of CSM patients, which proved impossible to expand despite considerable effort, primarily as a result of the strict inclusion criteria. At the beginning of the study, quite a large cohort of CSM patients being followed up in our department were screened for the presence of symptoms and signs similar to those seen in SFN. The prevalence of CSM, however, is significantly higher in older patients, among whom there are only a few individuals with a complete absence of risk factors for peripheral neuropathies together with normal large nerve‐fiber function according to nerve conduction studies. On the other hand, the inclusion criteria were strict enough to exclude as many patients with suspected coincidence of CSM and peripheral neuropathy (as a confounding factor for such a type of study) as possible.

Two of the CSM patients in this study showed borderline IENFD values according to our own reference data. Despite very strict inclusion criteria, the possible association of CSM and SFN cannot be excluded in these individuals. In a recent study, an overall minimum prevalence of SFN, about 52.95 per 100,000 individuals, was revealed, with an increasing incidence and prevalence in elderly patients compared with younger ones (Peters et al. 2013). SFN is thus not a rare condition, particularly in older adults, and one or two of our patients may be diagnosed with both. On the other hand, the IENFD results were only borderline in these cases and, when using the worldwide normative reference data, all values were within the normal range. Furthermore, most of the patients included in our study showed fully normal IENFD values according to both sets of reference data used. Considering their clinical symptoms and signs mimicking SFN, the study broadened a spectrum of controls for skin biopsy evaluation and provided further support for the use of IENFD assessment in painful peripheral neuropathies. IENFD is thus a valuable marker in the differentiation of peripheral neuropathy with small nerve‐fiber dysfunction from other possible causes of neuropathic pain in the feet, at both group and individual levels.

## Conflict of Interest

None declared.
